# Preliminary Evaluation of the Toxic Effects of Essential Oils as Natural Pesticides Against Maize Weevil (*Sitophilus zeamais*) and Its Fungal Pathogens

**DOI:** 10.3390/insects17010068

**Published:** 2026-01-06

**Authors:** Ompelege Jacqueline Phokwe, Kabelo Magoro, Mametsi Rahab Maseme, Madira Coutlyne Manganyi

**Affiliations:** 1 Department of Biological and Environmental Sciences, Faculty of Natural Sciences, Walter Sisulu University, Private Bag XI, Mthatha 5117, South Africa; ophokwe@wsu.ac.za; 2 Department of Biological and Environmental Sciences, Sefako Makgatho Health Sciences University, P.O. Box 139, Medunsa 0204, South Africa; 3 Department of Chemical and Physical Sciences, Walter Sisulu University, Private Bag XI, Mthatha 5117, South Africa; mmaseme@wsu.ac.za

**Keywords:** antifungal activity, botanical pesticides, *Eucalyptus globulus*, *Lantana camara*, sustainable pest management

## Abstract

To control maize weevils (*Sitophilus zeamais*), a major pest of stored grains, this study explores the use of essential oils from *Eucalyptus globulus* and *Lantana camara* as natural biopesticides. Given the risks of synthetic pesticides, these oils offer a sustainable alternative. The research first identified ten fungal pathogens associated with the weevils, including the dominant species, *Fusarium solani*. Preliminary results showed that both oils were then tested for their ability to kill the fungi and the weevils. *Eucalyptus globulus* oil proved to be a superior antifungal agent, inhibiting fungal growth by up to 93%, significantly outperforming *Lantana camara* oil. Both oils demonstrated potent insecticidal properties, achieving 100% weevil mortality at a 10% concentration within 24 hrs. However, *Eucalyptus* oil was more effective, maintaining 100% mortality even at a lower 5% concentration, unlike *Lantana* oil. Chemical analysis showed that *Eucalyptus* oil’s high effectiveness may be associated with its main component, eucalyptol (52.8%). *Lantana* oil had a more varied composition, with caryophyllene (31%) as its primary constituent. The findings suggest that *Eucalyptus globulus* essential oil is a promising, two-in-one biopesticide capable of controlling both maize weevils and their associated fungal pathogens.

## Simple Summary

Essential oils from eucalyptus (*Eucalyptus globulus*) and lantana (*Lantana camara*) were explored as sustainable, dual-action biopesticides against the maize weevil (*Sitophilus zeamais*) and its associated fungal pathogens. Both oils proved to be potent insecticides, achieving 100% weevil mortality at a 10% concentration within 24 hrs. However, eucalyptus oil was significantly more effective overall: it maintained 100% mortality even at a lower 5% concentration and acted as a superior antifungal agent, inhibiting fungal growth (including the dominant species, *Fusarium solani*) by up to 93%. Chemical analysis suggests this higher efficacy is attributable to its primary constituent, eucalyptol (52.8%), leading to the conclusion that *Eucalyptus globulus* essential oil is a promising natural control agent for both the weevils and the fungi they harbor.

## 1. Introduction

Ensuring food security for a rapidly expanding global population requires sustainable strategies that safeguard both productivity and environmental health [1,2,3]. One of the major threats to stored grain reserves is the maize weevil (*Sitophilus zeamais*), a destructive pest responsible for significant post-harvest losses worldwide [4,5]. While synthetic pesticides have effectively been employed to manage infestations, their continued use has led to widespread challenges, including ecological contamination [6,7], risks to human health [8,9], negative impacts on non-target organisms [10,11,12], and the emergence of resistant pest populations [13,14,15,16]. For instance, chemical insecticides such as malathion and chlorpyrifos often leave residue in grains [17], while fumigants like aluminium phosphide pose serious risks of toxicity through both ingestion and inhalation [5,18]. These limitations underline the need for safe, environmentally compatible pest control solutions.

Essential oils and other plant-derived bioactive compounds have gained increasing attention as viable alternatives to conventional pesticides. The pesticidal effects of essential oils are attributed to their complex secondary metabolites, including terpenoids, phenolics, flavonoids, and alkaloids [19,20,21,22], which display potent antimicrobial [23] and insecticidal properties [24,25]. Empirical evidence demonstrates their potential to suppress insect survival, reproduction, and feeding behaviour. For example, a study evaluated the insecticidal and repellent properties of free and microencapsulated *Cinnamomum cassia* essential oil (EO) against *Sitophilus zeamais* (maize weevil). The microencapsulated EO showed prolonged insecticidal activity, maintaining 45% efficacy on day 15 and remaining nearly stable for 65 days. The microcapsules also demonstrated reusability, retaining 70% mortality across five cycles, highlighting their potential as a sustainable approach to maize weevil management [26]. Similarly, *Apium graveolens* L. (celery) seed essential oil demonstrated potent insecticidal and repellent activities against the maize weevil, S. zeamais. Its effectiveness as a contact insecticide was dose- and time-dependent, with an LC_50_ of 19.83 nL/adult after 72 hrs. Furthermore, the oil exhibited strong repellent properties at concentrations above 16 µL/L of air, indicating its potential as a natural and sustainable alternative to synthetic pesticides [27].

A study by Yang et al. found that cinnamon oil was highly effective against *S. zeamais* via contact and fumigation, with its primary component being trans-cinnamaldehyde. However, the study noted its reduced efficacy in containers with grains, suggesting a need for controlled-release technologies to improve its persistence. Similarly, research by Pimenta et al. showed that clove essential oil and its main identified component, eugenol, had significant lethal, fumigant, and repellent effects. The oil was found to be highly toxic with an LC_50_ of 10.15 µL/g causing physiological and biochemical changes in the insect, highlighting its potential for pest control [28]. In addition to clove and cinnamon oils, other essential oils have shown promise. Several studies have evaluated the repellent properties of essential oils from different Eucalyptus species. For example, research by Ben Miri et al. found that the essential oils of *Eucalyptus camaldulensis* and *Eucalyptus citriodora* showed excellent repellent activity. Another study on *Eucalyptus globulus* essential oil by Ngongo-Kapenga et al. revealed a dose-dependent fumigant effect, with a volume of 30 µL causing the highest mortality [29]. Lastly, an investigation into oregano essential oil demonstrated its potent insecticidal activity. Its key identified active compounds, carvacrol and thymol, were found to be highly toxic and repellent, offering a promising, eco-friendly solution for pest management [29].

Species such as *L. camara* and *Eucalyptus globulus* are of particular interest, as their extracts demonstrate notable insecticidal and antifungal activities alongside a reduced environmental footprint [4,30,31]. Such findings highlight the efficacy of phytogenic pesticides, which are not only biodegradable but also exhibit broad-spectrum bioactivity with minimal ecological disruption. *Eucalyptus* essential oil (EO) is a promising alternative to synthetic pesticides because it is rich in bioactive compounds [32] which are reported to exhibit insecticidal, repellent, and antimicrobial properties [25]. Its biological activity is largely determined by the major terpene constituents α-pinene and β-caryophyllene [33]. Additionally, the global abundance of *Eucalyptus* biomass, high oil yields, and growing market demand make their large-scale extraction and application economically feasible. While *Lantana* oil is more locally abundant and underutilized, it offers multiple benefits as a pest control agent [34,35], owing to its diverse blend of bioactive compounds such as β-caryophyllene [36,37], α-pinene [38], and cadina-1(10),4-diene [39]. This complex phytochemical profile contributes to insecticidal, antifeedant, repellent, and antifungal activities, often through synergistic interactions among its constituents. 

This study evaluates the insecticidal and antifungal potential of *L. camara* and *E. globulus* essential oils against the maize weevil (*Sitophilus zeamais*) and its associated fungal pathogens. By identifying effective essential oil types and concentrations, this research contributes to the development of plant-based alternatives to synthetic pesticides, promoting sustainable pest management and reducing dependence on hazardous chemical practices. The efficacy of both essential oils was assessed through contact toxicity, repellence, and antifungal bioassays.

## 2. Materials and Methods

### 2.1. Ethical Approval

This study was conducted in accordance with ethical guidelines for research involving living organisms and was approved by the University Research Ethics Committee (UREC) of Walter Sisulu University, Mthatha Campus. Ethics clearance was granted under protocol number WSU/FNS-GREC/2024/03/11/G5, ensuring adherence to institutional and international ethical standards. All experimental procedures were designed to safeguard the welfare of biological specimens and to maintain the integrity of insect-handling protocols.

### 2.2. Collection of Maize Weevils

The initial stock of maize weevils (*Sitophilus zeamais*) was obtained from GoodUkhanyo Farmer Development, Mthatha, Eastern Cape, South Africa (31°35′19″ S, 28°47′24″ E). The insects were reared on untreated maize kernels purchased from a local store and maintained under controlled laboratory conditions of 28.6 ± 2 °C, 65.6 ± 5% relative humidity, and a 12:12 light:dark (L:D) photoperiod. The cultures were kept in aerated glass jars with perforated lids covered by nylon mesh to prevent insect escape while allowing ventilation. Stock cultures were regularly renewed to maintain healthy insect populations for bioassays.

### 2.3. Collection of Plant Materials

Fresh leaves of *L. camara* and *E. globulus* were collected from the O.R. Tambo District, KSD Region, Eastern Cape, South Africa (31.7074° S, 28.5798° E). The region experiences an annual average rainfall of ~117 mm, with notable seasonal variation. Plant specimens were identified and authenticated by Mr. Prinavin Naidu (Herbarium Curator) and Dr. Madeleen Struwig (Taxonomist), Department of Botany, North-West University. Voucher specimens were deposited in the Walter Sisulu University Herbarium.

### 2.4. Preparation of Essential Oils

Fresh leaves of *Lantana camara* and *Eucalyptus globulus* (200 g each) were used for essential oil extraction following the hydrodistillation method described by Atti-Santos et al. [40]. Plant material was placed in a 500 mL round-bottom flask and submerged in 2.5 L of distilled water. The flask was heated on a mantle at 160–170 °C and connected to a Clevenger-type apparatus with a condenser to facilitate oil collection. Distillation was maintained for 3 hrs, after which the essential oils were collected, dried over anhydrous sodium sulfate to remove residual moisture, and weighed. Oils were stored in airtight amber vials at 4 °C until use.

### 2.5. Isolation of Pathogenic Fungi

Adult maize weevils were surface-sterilized in 70% ethanol for 1–2 min, rinsed three times with sterile distilled water, and air-dried on sterile filter paper under a laminar flow hood (Laminar Airflow LLFV-204, Leicester, England, UK) Using sterile scalpels and forceps, insects were dissected, and internal tissues were aseptically transferred onto Potato Dextrose Agar (PDA, Merck Biolab, Johannesburg, Gauteng, South Africa) plates. The plates were sealed with Parafilm^®^ to prevent contamination and incubated (Scientific 160L Digital Incubator, Midrand, South Africa) at 25–28 °C in the dark for 10 days. Emerging fungal colonies were subcultured onto fresh PDA to obtain pure isolates.

### 2.6. Morphological Identification of Fungal Isolates

Fungal colonies were characterized based on macroscopic features, including colony color, texture, and growth patterns. Microscopic features were observed by preparing wet mounts in sterile distilled water and covering them with glass cover slips. Micromorphological traits such as spore shape, conidial arrangement, hyphal septation, and the presence of special structures (e.g., sclerotia, chlamydospores) were examined under a compound microscope (Nikon Eclipse Ni, Randburg, South Africa) and photomicrographs were taken for documentation. Preliminary identification was carried out using standard fungal taxonomic keys [41].

### 2.7. Molecular Identification of Fungal Isolates

#### 2.7.1. Genomic Deoxyribonucleic Acid (DNA) Extraction

Genomic DNA was extracted from pure fungal cultures using the ZR Fungal/Bacterial DNA MiniPrep Kit (Catalogue No. 06005, Inqaba Biotec, Pretoria, South Africa), following the manufacturer’s protocol as adapted from Gopane et al. [41].

#### 2.7.2. PCR Amplification

The internal transcribed spacer (ITS) region of rDNA was amplified using universal primers ITS1 and ITS4. PCR reactions (20 µL) consisted of 2 µL 10× buffer, 2 µL dNTP mix, 0.5 µL of each primer, 0.5 µL Taq polymerase, 9 µL nuclease-free water, and 5 µL template DNA. PCR cycling conditions were: initial denaturation at 94 °C for 5 min, followed by 35 cycles of denaturation at 94 °C for 30 s, annealing at 50 °C for 30 s, and extension at 68 °C for 1 min (Bio-Rad C1000 Touch^TM^ Thermal Cycler, Johannesburg, South Africa), with a final extension at 68 °C for 10 min.

#### 2.7.3. Agarose Gel Electrophoresis

PCR products were resolved on 1% agarose gel (CSL-AG500, Cleaver Scientific Ltd., Rugby, Warwickshire, UK) stained with EZ-Vision^®^ BlueLight DNA dye. DNA size was estimated using the NEB Fast Ladder (N3238; New England Biolabs, Ipswich, MA, USA).

#### 2.7.4. Sequencing and Data Analysis

PCR amplicons were purified using the ZR-96 DNA Sequencing Clean-up Kit™ (Zymo Research, Catalogue No. D4050, Inqaba, Pretoria, RSA) and sequenced bidirectionally using the ABI 3730xl Genetic Analyzer (Thermo Fisher Scientific, Foster City, CA, USA) with the BrilliantDye™ Terminator v3.1 Kit (Nimagen, Nijmegen, The Netherlands). Forward and reverse chromatograms were assembled into consensus sequences using CLC Bio Main Workbench veision 20.0.3. Sequences were analysed via BLAST version 2.16.0 against the NCBI GenBank database to confirm fungal identities.

### 2.8. Antifungal Activity of Essential Oils

The antifungal potential of *L. camara* and *E. globulus* essential oils was evaluated against ten fungal isolates obtained from *S. zeamais*. Fungal isolates were reactivated and maintained on Potato Dextrose Agar (PDA) until active mycelial growth was achieved. Spore suspensions were prepared by gently scraping 7-day-old cultures with sterile distilled water, filtering to remove hyphal fragments, and adjusting to a uniform inoculum density. For each treatment, 30 mL of molten PDA supplemented with 0.05% Tween 80 was mixed with 2 mL of the spore suspension and poured into sterile 90 mm Petri dishes. After solidification, sterile 6 mm paper discs were placed equidistantly on the agar surface and impregnated with 20 µL of essential oil at concentrations of 100, 250, 500, 1000, and 2000 µL/L to assess dose-dependent effects. Sterile distilled water-treated discs served as negative controls. No chemical positive control was used to allow direct comparison among essential oil treatments. A 5 mm diameter mycelial plug from the margin of an actively growing fungal colony was aseptically placed at the center of each plate. Plates were incubated at 25 ± 1 °C in the dark for 5–7 days, depending on species growth rate. Radial mycelial growth was measured using a ruler or digital caliper, and percentage inhibition was calculated according to Singh et al. [42]:

$$Inhibition(\%)=\frac{C-T}{C}\times 100$$

where: C = average radial growth in the control (mm); T = average radial growth in the treatment (mm)

### 2.9. Contact Toxicity Effect of Essential Oils Against Maize Weevil

The contact toxicity of *L. camara* and *E. globulus* essential oils against *Sitophilus zeamais* was evaluated using a topical application assay. Measured volumes of essential oil (0.5 mL and 1 mL) were evenly applied to 10 g of sterilized maize grains (*w*/*w*) and transferred into sterile Petri dishes. Five adult weevils (5–10 days old) were introduced per dish, and each treatment was replicated three times. Malathion-treated maize served as a positive control, while untreated maize served as a negative control. Mortality was recorded daily over a 10-day exposure period. Weevils were considered dead if no movement was observed when gently probed with a fine needle. The assay allowed assessment of dose-dependent insecticidal effects of essential oils in comparison to conventional chemical control.

### 2.10. Repellence Assay of Essential Oils Against Maize Weevil

Repellence of the essential oil against *Sitophilus zeamais* was evaluated using a zone-choice bioassay adapted from established protocols. The assay was performed in 9 cm diameter glass Petri dishes lined with bisected Whatman No. 1 filter paper. One half-disc was uniformly treated with 0.5 mL of essential oil solution at different concentrations (0.125, 0.25, 0.5, 1.0, and 2.0% *v*/*v* in acetone), while the other half-disc received 0.5 mL of pure acetone to serve as a negative control. Both halves were air-dried at room temperature for 15 min to allow for complete solvent evaporation before being placed adjacently inside the Petri dish. For each concentration, ten unsexed adult *S. zeamais* (2–3 weeks old) were carefully released at the centre of the dish. The experiment was conducted under controlled laboratory conditions (27 ± 2 °C, 65 ± 5% RH, and 12:12 hrs light:dark photoperiod). The number of insects on each half of the filter paper was recorded at 1, 2, 4, 6, 12, and 24 hrs after exposure. The percentage repellence (PR) was calculated for each concentration and exposure time using the following formula:

$$PR=\frac{N_{c}-N_{t}}{N_{c}+N_{t}}\;\times 100$$

where *N_c_* = number of insects on the control half and *N_t_* = number of insects on the treated half.

Repellence was further classified into six standard categories (Class 0: 0.01–0.1%, Class I: 0.1–20%, Class II: 20.1–40%, Class III: 40.1–60%, Class IV: 60.1–80%, and Class V: 80.1–100%) to determine the degree of effectiveness. Each treatment was replicated five times, and the mean repellence values were subjected to statistical analysis to assess concentration- and time-dependent effects.

### 2.11. GC-MS Analysis of Essential Oils

The chemical composition of *Lantana camara* and *Eucalyptus globulus* essential oils was determined using gas chromatography–mass spectrometry (GC–MS) at the Department of Microbial, Biochemical & Food Biotechnology, University of the Free State, South Africa. Analyses were performed on a Thermo Scientific Trace 1310 gas chromatograph equipped with a flame ionization detector (FID) and coupled to a Thermo Scientific ISQ 7000 single quadrupole mass spectrometer. Separation was achieved using an SGE BP5MS capillary column (60 m × 0.25 mm i.d., 0.25 µm film thickness). Hydrogen was used as the carrier gas at a constant pressure of 200 kPa. The injector and FID were maintained at 290 °C, the transfer line at 280 °C, and the ion source at 200 °C. The oven program was set at 60 °C (10 min hold), ramped at 5 °C/min to 300 °C, and held for 30 min. The MS was operated in scan mode over the range 45–650 m/z. Instrument control and data acquisition were carried out using Thermo Xcalibur 4.0 software. Essential oil samples were diluted in 1 mL of the extraction solvent prior to analysis. Compounds were identified by comparing the electron ionization mass spectra with those in the NIST 2017 library. Authentic standards were not available for all compounds; therefore, identifications are reported as tentative and are supported by manual evaluation of fragmentation patterns and consistency with previously reported constituents of *L. camara* [43,44,45] and *E. globulus* [32,46,47] essential oils. Only constituents present at ≥1% of the total composition were included, while trace components below this threshold were excluded due to their negligible contribution.

### 2.12. Statistical Analysis

Data were analysed using one-way Analysis of Variance (ANOVA), and differences among treatment means were compared using Tukey’s post hoc test at a significance level of *p* < 0.05. Results are presented as mean ± standard deviation (SD).

The mean (μ) for each treatment was calculated as: $\sigma =\;\sqrt{\frac{\sum {\left(\mu_{i}-\;\mu \right)}^{2}}{N-1}}$. The mean value, μ, was calculated using the formula: $\mu =\;\frac{\sum_{i=1}^{N}\mu_{i}}{N}$ where N represents the number of plates that make up a specific isolate and $\mu_{i}$ is the measured diameter on each plate.

## 3. Results

### 3.1. Morphological Identification of Fungal Pathogens

**Figure 1:**
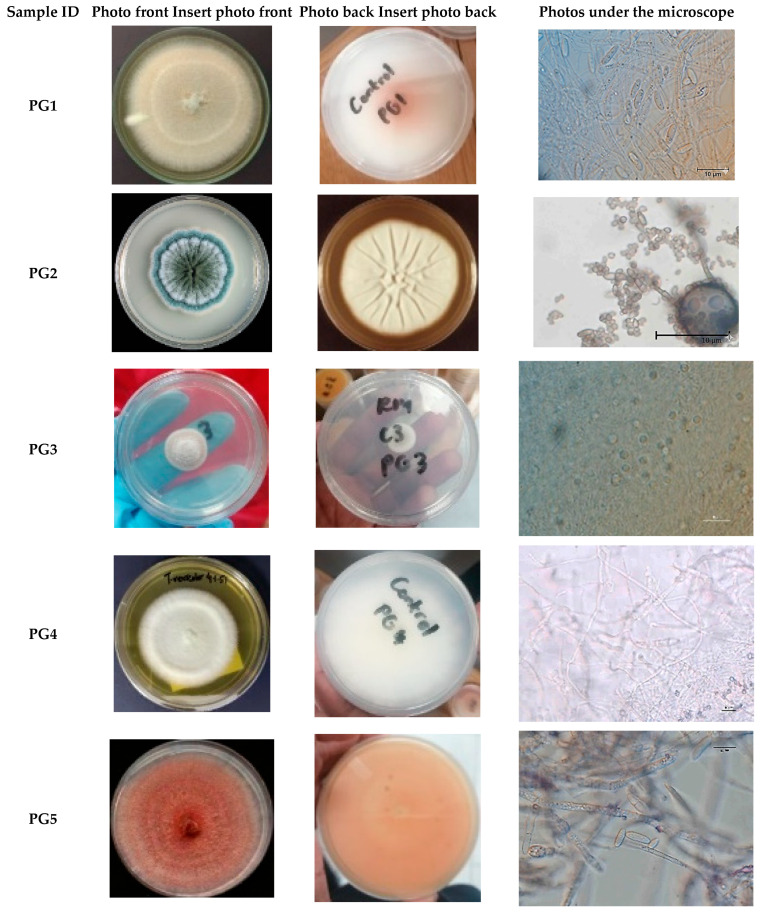

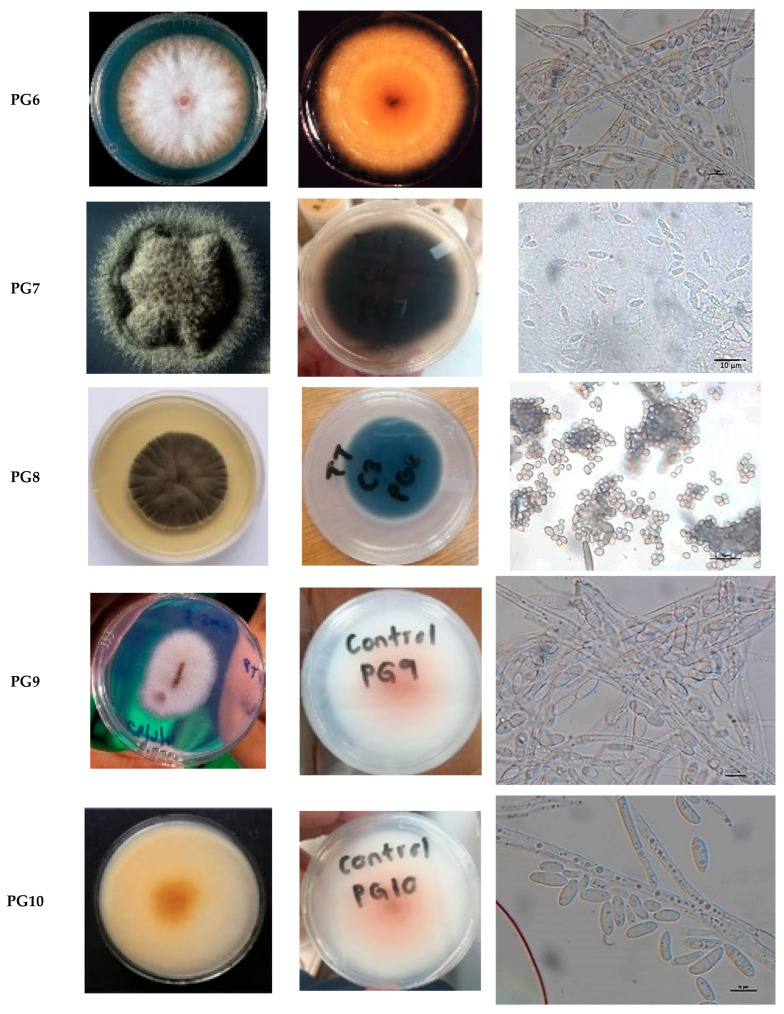
Visual and Microscopic Characterization of Pathogenic Fungi, Including Colony colour.

A total of ten fungal isolates (n = 10) were recovered from maize weevils (*Sitophilus zeamais*) infesting maize (*Zea mays*), representing diverse genera, including *Fusarium*, *Penicillium*, *Purpureocillium*, *Cladosporium*, and *Trametes*. As shown in Table 1 and Figure 1, the isolates PG1, PG6, PG9, and PG10 displayed white colonies on the frontal surface and orange-white pigmentation on the posterior surface, with filamentous, radially fuzzy margins. PG5, identified as *Fusarium*, exhibited pink colonies front and back, with septate hyphae, oval spores, and sickle-shaped conidia. PG2 and PG8 formed velvety, green colonies with undulating or lobed margins; microscopic examination revealed aerial hyphae, oval spores, and penicillate conidiophores, confirming *Penicillium* identification. PG3 presented light purple and white frontal colonies with cream-white posterior surfaces; hyaline, septate hyphae, ellipsoidal spores, and phialides supported classification as *Purpureocillium*. PG7 produced black-brown filamentous colonies with radial fuzzy margins, containing lemon-shaped spores and shielded conidiophores, consistent with *Cladosporium*. PG4 exhibited white filamentous colonies with clamp connections and smooth cylindrical spores, lacking conidiophores, and was identified as *Trametes*. Overall, morphological characterization revealed considerable diversity, with *Fusarium* being the most prevalent genus among the isolates.

### 3.2. Molecular Identification of Fungal Pathogens

Molecular techniques were employed to confirm the identification of fungal isolates by comparing their sequences with those stored in the GenBank database (Table 2). Isolate PG1, PG5, PG6, PG9, and PG10 were all identified as *Fusarium solani* with the accession number OQ818134.1, demonstrating the abundance of this pathogen among the samples. This highlights *F. solani* as the dominant species in the sampled environment. Similarly, isolate PG2 was identified as *Penicillium brasilinum* with the accession number AB455514.2. Further analysis showed that isolate PG3 was *Purpureocillium* (accession number: MK503783.1). Isolate PG4 was identified as *Trametes versicolor* (accession number OR250362.1), and isolate PG7 was identified as *Cladosporium* sp. (accession number OP596126.1). The identification of isolate PG8 as *Penicillium* sp. (accession number MN788660.1) highlights the presence of another distinct *Penicillium* species. These findings provide valuable insights into the fungal biodiversity and potential ecological interactions occurring within the sampled environment.

### 3.3. Antifungal Activity of Essential Oils Against Fungal Pathogens from S. zeamais

The antifungal potential of *L. camara* and *E. globulus* essential oils was evaluated against ten fungal isolates obtained from *S. zeamais* (Table 3). Both oils demonstrated concentration-dependent inhibition, with efficacy increasing at higher concentrations (100–2000 µL/L). Among the isolates, PG5, PG8, and PG10 exhibited higher resistance at lower doses but increased susceptibility at higher concentrations. Several isolates, including PG2, PG3, PG6, and PG7, were highly sensitive to *E. globulus* essential oil. At the highest concentration (2000 µL/L), inhibition reached up to 93%, with PG3 showing 90% growth suppression. Comparative analysis revealed that *E. globulus* essential oil consistently outperformed *L. camara* oil across most concentrations, particularly at 1000 and 2000 µL/L. Both oils demonstrated fungistatic or fungicidal properties, with enhanced inhibitory effects at higher doses. *L. camara* oil was effective but generally required higher concentrations to achieve inhibition levels comparable to *E. globulus*, suggesting slightly lower broad-spectrum antifungal activity. Overall, these findings underline the superior efficacy of *E. globulus* essential oil as a potential biopesticide with both insecticidal and antifungal properties.

### 3.4. Contact Toxicity Effect of Essential Oils Against Maize Weevil

**Figure 2:**
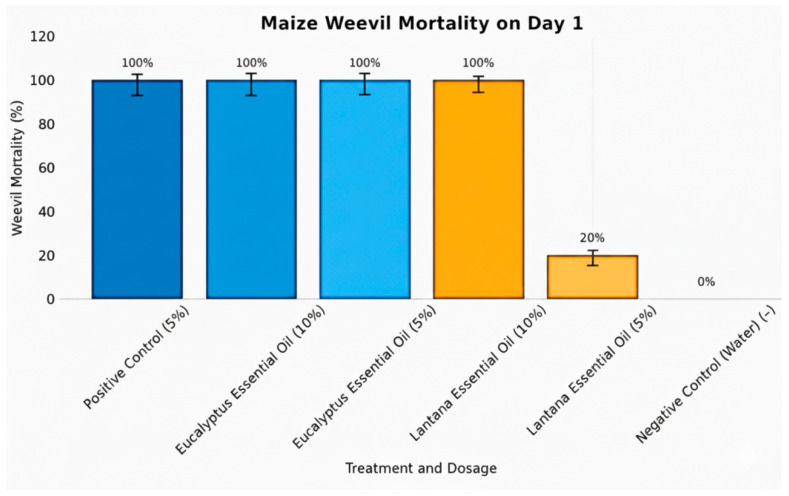
Graph showing contact toxicity of essential oils against maize weevils.

The insecticidal efficacy of *E. globulus* and *L. camara* essential oils against *Sitophilus zeamais* was monitored over a 10-day period (Table 4 and Figure 2). Preliminary results showed that positive control (malathion, 5%) caused immediate and complete mortality, achieving 100% death within 24 hrs. The negative control (distilled water) exhibited no mortality. Both concentrations of *E. globulus* essential oil (5% and 10%) demonstrated rapid and potent insecticidal effects, achieving complete weevil mortality (100%) within 24 hrs. Mean mortality values were 100 ± 4.5% for the 10% concentration and 100 ± 3.0% for the 5% concentration, suggesting strong toxicity regardless of concentration. In contrast, *L. camara* essential oil exhibited a concentration-dependent response. At 10%, the oil induced 100 ± 1.2% mortality on Day 1, comparable to both the positive control and *E. globulus* oil. However, the 5% concentration showed reduced and inconsistent efficacy, with mortality ranging from 20 ± 3.2% on Day 1 to 40 ± 0.2% on Day 6, and zero mortality on several intermediate days. These results indicate that *L. camara* oil requires threshold concentration to achieve optimal insecticidal activity, whereas *E. globulus* oil is highly effective even at lower concentrations.

### 3.5. Repellence Bioassay

**Figure 3:**
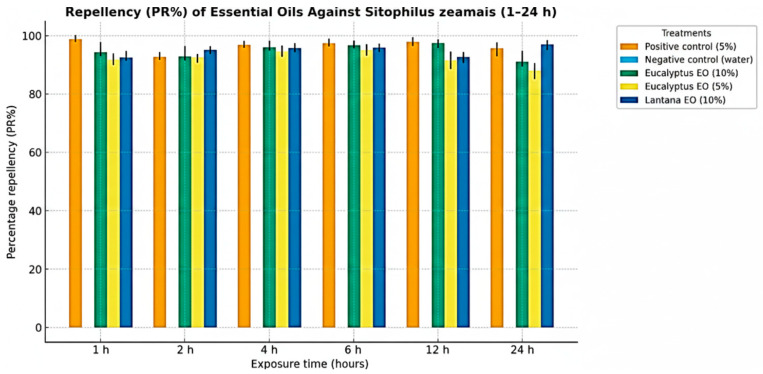
Bar graph showing the repellence (PR%) of essential oils against *Sitophilus zeamais* across the 1–24 h exposure intervals.

Preliminary results of repellence bioassay revealed clear concentration- and plant species–dependent differences in the behavioral response of *S. zeamais* adults to essential oil treatments (Table 5 and Figure 3). The positive control (synthetic repellent, 5%) achieved complete repellence (100 ± 2.0%), while no repellence was observed in the negative control (water) throughout the trial (0%). Among the test oils, *E. globulus* oil exhibited strong and consistent activity at both 10% and 5% concentrations, with mean repellence values exceeding 95% within the first hour of exposure and remaining in repellence Class V (80-100%) for up to 24 h. This demonstrates that *E. globulus* oil is a highly effective repellent even at moderate dosages. *L. camara* essential oil also showed high repellence at 10% concentration, producing a repellence level of 100 ± 1.2% on Day 1, consistent with Class V activity. In contrast, the 5% concentration of *L. camara* oil displayed variable and generally weak effects, with repellence fluctuating between 20 and 40% across observation days and remaining within Classes I–II. Overall, *E. globulus* oil demonstrated significantly higher and more stable repellence than *L. camara* oil at equivalent concentrations (*p* < 0.05). The effect of concentration was also statistically significant (*p* < 0.001), with higher dosages producing markedly stronger repellence.

### 3.6. GC-MS Analysis 

**Figure 4:**
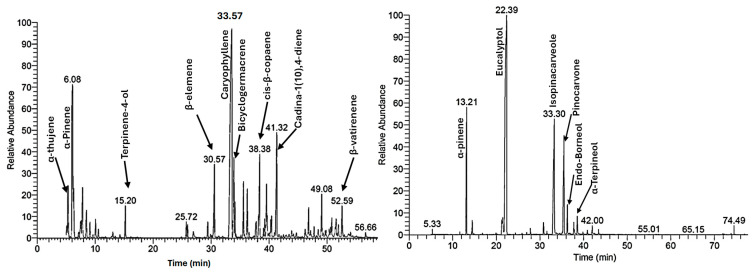
GC-MS chromatogram of essential oils extracted from *L. camara* (**left**) and *E. globulus* (**right**) leaves.

Gas chromatography-mass spectrometry (GC-MS) analysis of the essential oils from *E. globulus* and *L. camara* species revealed distinct phytochemical profiles, with each oil containing a unique blend of compounds as shown in Figure 4. The chemical compounds with significant abundance (≥1%) identified in the essential oil of *L. camara* and *E. globulus* leaves, along with their relative percentages, are presented in Table 6, while a detailed table including their MS fragmentation ions and selected mass spectra is provided in Table S1 and Figures S1–S8 (Supplementary Data). The analysis of *L. camara* exhibited a sesquiterpene-dominant chemical profile. The most abundant compound was identified as caryophyllene (30.99%), followed by cis-β-copaene (5.02%), cadina-1(10),4-diene (5.92%), bicyclogermacrene (4.11%), and β-elemene (4.08%). The high content of caryophyllene is known to contribute to the oil’s antimicrobial and anti-inflammatory properties [19]. α-pinene (13.83%) was identified as the most abundant monoterpenes. In contrast, *E. globulus* oil revealed a cineole-rich chemotype, with eucalyptol (1,8-cineole, 52.77%) as the major constituent. This was found alongside monoterpene hydrocarbon α-pinene (9.09%) and oxygenated monoterpenes isopinocarveole (16.72%) and pinocarvone (10.93%). This composition is consistent with literature reports that consistently identify *E. globulus* oil as cineole-dominant, typically containing approximately 17–90% eucalyptol [20].

## 4. Discussion

The present study successfully identified a diverse range of fungal pathogens, including *Fusarium*, *Penicillium*, *Purpureocillium*, *Cladosporium*, and *Trametes*, associated with maize weevils (*Sitophilus zeamais*). The prevalence of *Fusarium* species, particularly *Fusarium solani* (isolates PG1, PG5, PG6, PG9, and PG10), as the dominant pathogen is a notable finding. This result is consistent with existing literature, which frequently reports *Fusarium* species as significant post-harvest pathogens of maize [48,49,50]. In a separate but related discovery, a recent academic paper reported the first-ever isolation of the yeast *Hyphopichia burtonii* from the storage pest, *S. zeamais*. This novel finding is significant because the isolated yeast demonstrated strong bioactivity against mycotoxigenic fungi. The isolation suggests that the weevil may serve as a vector for beneficial microorganisms that could be leveraged for the biological control of fungal contamination in stored grains [51]. The molecular identification of all isolates, which confirmed the morphological findings, is crucial, as morphological characteristics alone can sometimes be ambiguous. The identification of PG4 as *Trametes versicolor*, a white-rot fungus, highlights the unexpected biodiversity associated with the weevil’s ecosystem.

The antifungal potential of the essential oils was also found to be concentration-dependent, with *E. globulus* oil demonstrating superior broad-spectrum activity compared to *L. camara* oil. The high level of inhibition observed with *E. globulus* oil is consistent with numerous studies highlighting the potent fungicidal properties of its major components, eucalyptol and α-Pinene. These compounds are thought to disrupt fungal cell membranes, leading to cell lysis and growth inhibition by increasing membrane permeability [23,52,53]. The observed resistance of certain isolates, such as PG5 (*Fusarium solani*), PG8 (*Penicillium* sp.), and PG10 (*Fusarium solani*), to the essential oils at lower concentrations is a critical finding. The concept of concentration-dependent efficacy is a cornerstone of antifungal research. Numerous studies have established that essential oils often require a minimum inhibitory concentration (MIC) to be effective [54,55]. For instance, research on *Eucalyptus camaldulensis* essential oil found that while it could inhibit *F. solani* at lower concentrations, the effectiveness was significantly lower at reduced doses [56]. Similarly, other research has highlighted the variability in resistance among different strains of the same fungal species. This suggests that the genetic and physiological differences between individual strains can influence their susceptibility, a factor that our finding of resistance in specific isolates (PG5, PG8, PG10) strongly supports. On the other hand, Wu et al. demonstrated that *Sclerotinia sclerotiorum* is the most resistant pathogen to the essential oils tested, while *Rhizoctonia solani* is the most susceptible. The resistance of *S. sclerotiorum* is evident from its consistently higher EC_50_s, while the susceptibility of *R. solani* is evidenced by its consistently low EC_50_s across treatments. This aligns with the idea that the essential oils are not universally effective at all concentrations and that certain fungal isolates are better equipped to withstand their effects. The mechanisms behind this resistance are also a topic of extensive study, providing context for the observed results. Fungi can defend against essential oils through several pathways, including having a more robust cell wall, possessing efflux pumps that actively expel the oil’s active compounds, or producing enzymes that metabolize and detoxify the antifungal compounds [57,58,59]. This highlights why the effectiveness of essential oils against fungi is a complex biological interaction. Moreover, it explains why efficacy can vary so widely and why higher concentrations or combinations of essential oils are often required to overcome these cellular-level defences.

The insecticidal activity of both *E. globulus* and *L. camara* essential oils against *S. zeamais* was confirmed to be rapid and potent. The 100% mortality observed within 24 hrs for both *Eucalyptus* (at 5% and 10%) and *L. camara* (at 10%) is particularly significant and can be explained by their compositional differences. The immediate and complete mortality from *E. globulus* oil can be directly linked to its dominant component, eucalyptol (1,8-cineole), a well-documented insecticidal agent against stored-product pests [32,60,61,62]. The fumigant action of eucalyptol is believed to be neurotoxic, affecting the insect’s octopaminergic nervous system, leading to overstimulation, paralysis, and death [18,63,64]. In contrast, the insecticidal effect of the diverse blended *L. camara* oil is likely due to the synergistic action of its key constituents, caryophyllene and α-Pinene, as well as the minor compounds [65]. These terpenoids possess insect-repellent and toxic properties, also acting as neurotoxins by disrupting the central nervous system [22,66]. The slow concentration-dependent response of *L. camara* oil, where the 5% concentration was largely ineffective, suggests a minimum threshold is required for its bioactive compounds to exert a lethal effect.

In addition to their insecticidal and antifungal effects, the present study also demonstrated the strong repellence activity of *E. globulus* and *L. camara* essential oils against *S. zeamais*. The repellence bioassay revealed that both concentrations of *E. globulus* oil (5% and 10%) produced consistent and sustained repellence responses, with percentage repellence values exceeding 95% across all observation intervals. These results place *E. globulus* oil within repellence Class V, confirming its potency as a behavioral deterrent. The high repellence activity is attributable to the volatility of eucalyptol, which readily disperses in closed storage environments and interferes with the chemosensory mechanisms of insects, thereby discouraging contact with treated substrates. *L. camara* oil also exhibited strong repellence at 10%, achieving comparable deterrent effects to those of *E. globulus* oil. However, the 5% concentration displayed inconsistent repellence, fluctuating between Classes I and II. This irregular activity suggests that a threshold concentration is required for the volatiles in *L. camara* oil to accumulate in sufficient amounts to induce avoidance behaviour. The significantly higher and more stable repellence of *E. globulus* oil compared to *L. camara* at equivalent concentrations emphasises its greater utility as a botanical repellent.

Our findings show that a single, cineole-rich *Eucalyptus globulus* oil achieved rapid weevil mortality and up to 93% inhibition of dominant fungi such as *Fusarium solani*, outperforming many single-target botanical treatments reported previously [26,28,29,55,67]. This dual efficacy offers a significant management advantage for smallholder grain storage, where fungal contamination and insect infestation typically occur together and chemical fumigants pose health and resistance risks. Integrating such broad-spectrum plant-based biopesticides into post-harvest systems could reduce chemical pesticide reliance while addressing two critical loss pathways: mycotoxin-producing fungi and insect damage in a single, environmentally compatible intervention.

## 5. Conclusions

In conclusion, the preliminary results demonstrate that both *E. globulus* and *L. camara* essential oils hold significant promise as biopesticides for the management of *S. zeamais* and its associated fungal pathogens. The two essential oils exhibit distinct chemotypes. While *L. camara* oil is sesquiterpene-rich, dominated by β-caryophyllene and supplemented by α-pinene and other sesquiterpenes, cineole-rich *E. globulus* oil contains eucalyptol as major component with significant amounts of isopinocarveole, pinocarvone, and α-pinene. The presence of these components supports the potential antimicrobial and insecticidal activities of the oils. The efficacy of *L. camara* oil relies on the synergistic interactions of multiple constituents, requiring higher doses to reach toxic thresholds, while the activity of *E. globulus* is driven by a single dominant compound with rapid action. However, the varying levels of resistance among the fungal isolates highlight the need for further research into the specific mechanisms of action and potential for adaptation. Future studies should focus on the synergistic effects of the oil components and their long-term efficacy in a stored grain environment.

## Figures and Tables

**Table 1:** Morphological Identification of Pathogenic Fungi: Macroscopic and Microscopic Characteristics.

Sample ID	Colony Colour (Front)	Colony Colour (Back)	Colony Texture	Colony Margin	Nature of Hyphae	Spores and Conidiophores	Probable ID
**PG1**	White	Orange and white	Filamentous	Radial and Fuzzy	Septate	Oval spores and sickle-shaped conidia	*Fusarium*
**PG2**	Dark Green with a white border	Green	Velvety	Wavy	Aerial	Oval spores and Penicillate conidiophores	*Penicillium*
**PG3**	Light purple and white	Cream white	Velvety	Smooth	Hyaline and Septate	Ellipsoidal spores with phialides terminating conidiophores	*Purpureocillium*
**PG4**	White	White	Filamentous	Radial and Fuzzy	Septate hyphae with clamp connections	Smooth cylindrical spores and no conidiophores	*Unknown*
**PG5**	Pink	Pink	Filamentous	Radial and Fuzzy	Septate	Oval spores and sickle-shaped conidia	*Fusarium*
**PG6**	Cream white	Red-Orange centre with white	Filamentous	Radial and Fuzzy	Septate	Oval spores and sickle-shaped conidia	*Fusarium*
**PG7**	Black or brown	Black or brown	Filamentous	Radial and Fuzzy	Septate	Lemon shaped spores and conidiophores with shield cells	*Cladosporium*
**PG8**	Military green	Military green	Velvety	Lobed	Aerial	Oval spores and Penicillate conidiophores	*Penicillium*
**PG9**	Red line surrounded by cream white	Red line surrounded by cream white	Filamentous	Radial and Fuzzy	Septate	Oval spores and sickle-shaped conidia	*Fusarium*
**PG10**	Orange centre surrounded by cream white	Orange centre surrounded by cream white	Filamentous	Radial and Fuzzy	Septate	Oval spores and sickle-shaped conidia	*Fusarium*

**Table 2:** Molecular Identification of fungal communities associated with *S. zeamais*.

Sample ID	Closest Related Species	GenBank Best BLAST Match		
		GenBank Accession	HSP Length	% Identity
PG1	*Fusarium solani*	OQ818134.1	569 bp	100%
PG2	*Penicillium brasilinum*	AB455514.2	593 bp	100%
PG3	*Purpureocillium lilacinum*	MK503783.1	604 bp	100%
PG4	*Trametes versicolor*	OR250362.1	630 bp	100%
PG5	*Fusarium solani*	OQ818134.1	568 bp	100%
PG6	*Fusarium solani*	OQ818134.1	515 bp	100%
PG7	*Cladosporium* sp.	OP596126.1	552 bp	100%
PG8	*Penicillium* sp.	MN788660.1	139 bp	100%
PG9	*Fusarium solani*	OQ818134.1	569 bp	100%
PG10	*Fusarium solani*	OQ818134.1	569 bp	100%

**Table 3:** Antifungal activity of *Eucalyptus globulus* and *Lantana camara* essential oils against pathogenic fungi from *S. zeamais*.

Plants	Fungal Pathogens	Inhibition (%) Concentrations				
		100 µL/L	250 µL/L	500 µL/L	1000 µL/L	2000 µL/L
**Lantana oil**	PG1	72 ± 1.2 ^a^	78 ± 0.7 ^a^	78 ± 0.9 ^a^	89 ± 0.0 ^e^	90 ± 1.2 ^e^
	PG2	81 ± 8.2 ^a^	85 ± 0.0 ^b^	89 ± 2.9 ^b^	89 ± 0.1 ^e^	89 ± 0.6 ^e^
	PG3	80 ± 2.1 ^b^	81 ± 0.3 ^b^	83 ± 0.2 ^b^	87 ± 0.4 ^b^	89 ± 0.0 ^e^
	PG4	67 ± 0.0 ^c^	72 ± 1.0 ^a^	83 ± 0.0 ^b^	83 ± 1.0 ^b^	85 ± 0.1 ^b^
	PG5	58 ± 0.7 ^d^	75 ± 0.4 ^a^	80 ± 2.4 ^b^	83 ± 0.7 ^b^	84 ± 0.7 ^b^
	PG6	80 ± 2.3 ^b^	81 ± 5.0 ^a^	82 ± 0.0 ^b^	83 ± 0.3 ^b^	89 ± 0.0 ^e^
	PG7	76 ± 0.4 ^a^	76 ± 0.6 ^a^	78 ± 0.4 ^a^	81 ± 2.4 ^f^	83 ± 0.9 ^b^
	PG8	65 ± 0.0 ^c^	71 ± 3.7 ^c^	82 ± 8.0 ^e^	82 ± 0.4 ^b^	88 ± 3.3 ^e^
	PG9	69 ± 0.1 ^c^	75 ± 0.0 ^a^	75 ± 0.6 ^a^	81 ± 0.9 ^b^	88 ± 0.1 ^b^
	PG10	67 ± 0.2 ^c^	72 ± 0.8 ^a^	78 ± 0.9 ^a^	78 ± 0.0 ^a^	83 ± 0.5 ^b^
**Eucalyptus oil**	PG1	71 ± 0.9 ^a^	72 ± 0.2 ^a^	73 ± 0.7 ^c^	75 ± 1.4 ^b^	83 ± 0.0 ^b^
	PG2	81 ± 0.1 ^b^	83 ± 0.5 ^b^	87 ± 2.3 ^d^	89 ± 0.8 ^d^	89 ± 0.6 ^d^
	PG3	83 ± 0.6 ^b^	83 ± 0.0 ^b^	87 ± 1.1 ^d^	87 ± 0.9 ^d^	90 ± 3.4 ^d^
	PG4	72 ± 3.3 ^a^	78 ± 3.2 ^c^	78 ± 0.8 ^e^	79 ± 0.3 ^b^	83 ± 0.9 ^b^
	PG5	71 ± 2.0 ^a^	75 ± 1.0 ^c^	76 ± 0.4 ^b^	83 ± 0.1 ^b^	83 ± 0.2 ^b^
	PG6	81 ± 0.9 ^b^	81 ± 0.0 ^b^	89 ± 0.4 ^d^	89 ± 0.9 ^f^	93 ± 0.0 ^f^
	PG7	82 ± 0.2 ^b^	82 ± 0.0 ^b^	88 ± 1.3 ^d^	89 ± 1.7 ^d^	89 ± 0.7 ^d^
	PG8	76 ± 1.5 ^c^	76 ± 0.4 ^c^	82 ± 0.9 ^b^	82 ± 0.0 ^b^	82 ± 1.0 ^b^
	PG9	69 ± 1.9 ^a^	75 ± 2.2 ^c^	75 ± 0.6 ^b^	81 ± 0.6 ^b^	81 ± 0.5 ^b^
	PG10	73 ± 0.6 ^a^	83 ± 0.3 ^b^	83 ± 0.3 ^b^	83 ± 0.8 ^b^	83 ± 0.1 ^b^

Tukey’s Honestly Significant Difference (HSD) post hoc test to determine significant differences between treatment means. Means labeled with different superscript lowercase letters are statistically different at *p* < 0.05.

**Table 4:** Percentage mean mortality of maize weevils treated with essential oils over 10 days.

Treatment	Dosage	Day 1	Day 2	Day 3	Day 4	Day 5	Day 6	Day 7	Day 8	Day 9	Day 10
Positive Control	5%	100 ± 2.0 ^a^	–	–	–	–	–	–	–	–	–
Negative Control (Water)	–	0	0	0	0	0	0	0	0	0	0
*Eucalyptus* Essential Oil	10%	100 ± 4.5 ^a^	–	–	–	–	–	–	–	–	–
*Eucalyptus* Essential Oil	5%	100 ± 3.0 ^a^	–	–	–	–	–	–	–	–	–
*Lantana* Essential Oil	10%	100 ± 1.2 ^a^	–	–	–	–	–	–	–	–	–
*Lantana* Essential Oil	5%	20 ± 3.2 ^b^	20 ± 2.^6 a^	0	20 ± 1.8 ^a^	0	40 ± 0.2 ^b^	0	0	0	0

Percentage mean mortality (Mean ± SD) of *Sitophilus zeamais* treated with essential oils over a 10-day period. Different superscript letters within the same column indicate significant differences among treatments based on one-way ANOVA followed by Tukey’s HSD test (*p* ≤ 0.05). Positive control = standard synthetic pesticide; Negative control = water.

**Table 5:** Percentage repellence (PR%) and corresponding repellence classes of essential oils against *Sitophilus zeamais* adults at different time intervals (1–24 h).

Treatment (Conc.)	1 h PR (%)	2 h PR (%)	4 h PR (%)	6 h PR (%)	12 h PR (%)	24 h PR (%)
Positive control (5%)	98.0 ± 1.2 ^a^	98.5 ± 0.9 ^b^	99.0 ± 0.7 ^a^	98.8 ± 0.8 ^a^	98.3 ± 1.0 ^a^	98.0 ± 1.1 ^a^
Negative control (water)	0.0 ± 0.0 ^c^	0.0 ± 0.0 ^c^	0.0 ± 0.0 ^c^	0.0 ± 0.0 ^c^	0.0 ± 0.0 ^c^	0.0 ± 0.0 ^c^
*Eucalyptus* EO (10%)	97.5 ± 1.5 ^a^	98.2 ± 1.1 ^a^	98.7 ± 0.9 ^a^	98.1 ± 1.0 ^a^	97.9 ± 1.3 ^a^	97.4 ± 1.4 ^a^
*Eucalyptus* EO (5%)	95.6 ± 1.9 ^a^	96.2 ± 1.7 ^a^	96.9 ± 1.6 ^a^	96.3 ± 1.8 ^a^	95.8 ± 2.0 ^a^	95.1 ± 2.1 ^a^
*Lantana* EO (10%)	96.9 ± 1.4 ^a^	97.3 ± 1.2 ^a^	97.8 ± 1.1 ^a^	97.1 ± 1.3 ^a^	96.6 ± 1.6 ^a^	96.0 ± 1.8 ^a^
*Lantana* EO (5%)	21.8 ± 2.9 ^b^	20.4 ± 2.6 ^b^	2.1 ± 1.0 ^b^	19.7 ± 2.5 ^b^	1.6 ± 0.9 ^b^	39.6 ± 2.1 ^b^

Percent repellency (Mean ± SD) of *Sitophilus zeamais* exposed to essential oils at different time intervals. Means followed by different superscript letters within the same column are significantly different (*p* ≤ 0.05) according to Tukey’s HSD test. Positive control = standard synthetic repellent; Negative control = water. (PR: percent repellency; EO: essential oil).

**Table 6:** Chemical composition of essential oil extracted from *L. camara* and *E. globulus* leaves. Only constituents with relative abundance ≥1% are included.

Identified Compound	Peak Area%	Identified Compound	Peak Area%
*L. camara*		*E. globulus*	
α-thujene	4.02	α-Pinene	9.09
α-Pinene	13.83	p-cymene	1.17
Camphene	1.99	m-cymene	1.86
Sabinene	1.55	Eucalyptol	52.77
β-Pinene	2.73	Isopinocarveole	16.72
β-Myrcene	1.47	Pinocarvone	10.93
Terpinene-4-ol	1.25	Endo-Borneol	1.65
β-elemene	4.08	α-Terpineol	1.14
Caryophyllene	30.99		
Bicyclogermacrene	4.11		
Alloaromadendrene	1.54		
(E)-β-Farnesene	2.26		
α-humulene	2.18		
γ-Curcumene	1.19		
cis-β-copaene	5.02		
γ-muurolene	3.04		
Cadina-1(10),4-diene	5.92		
Caryophyllene oxide	1.33		
Unknown *	1.98		
δ-Cadinene	1.36		
β-vatirenene	1.7		

* Mass spectral data did not allow reliable identification.

## Data Availability

The data supporting the findings of this study, including the raw and processed data for insect mortality and fungal inhibition assays, are available from the corresponding authors upon reasonable request. A detailed essential oil chemical composition analysis (quantified constituents, retention times and ion fragments) and selected mass spectra are available in Supplementary Data, Table S1 and Figures S1–S8, respectively.

## Supplementary Materials

The following supporting information can be downloaded at: https://www.mdpi.com/article/10.3390/insects17010068/s1, Figures S1–S8: MS spectra for compounds detected in *L. camara* and *E. globulus*; Table S1: Retention times, MS ion fragments and identified compounds for *L. camara* and *E. globulus* essential oils.
