# Supplementary material for: Preliminary Evaluation of the Toxic Effects of Essential Oils as Natural Pesticides Against Maize Weevil (Sitophilus zeamais) and Its Fungal Pathogens
Source: Insects. 2026 Jan 6;17(1):68. doi: 10.3390/insects17010068 (PMC12842577; doi:10.3390/insects17010068)
Supplement: Supplementary file 1 [file insects-17-00068-s001.zip › insects-3908658-supplementary.pdf]

Article

Evaluating the Toxic Effects of Essential Oils as Natural Pesticides Against Maize Weevil (*Sitophilus zeamais*) and Its Fungal Pathogens

Ompelege J. Phokwe [1], Kabelo Magoro[2], Mametsi R. Maseme [3] and Madira C. Manganyi [2,*]

[1] Department of Biological and Environmental Sciences, Faculty of Natural Sciences, Walter Sisulu University, Private Bag XI, Mthatha 5117, South Africa
[2] Department of Biological and Environmental sciences, Sefako Makgatho Health Sciences University, P.O. Box 139, Medunsa 0204, South Africa
[3] Department of Chemical and Physical Sciences, Walter Sisulu University, Private Bag XI, Mthatha 5117, South Africa
* Correspondence: madira.manganyi@smu.ac.za; madiramanganyi@gmail.com

Supplementary Data

Contents:

Table S1. Retention times, MS ion fragments and identified compounds for *L. camara* and *E. globulus* essential oils.
Figure S1: MS spectra for α-Pinene detected in *L. camara.*
Figure S2: MS spectra for Caryophyllene detected in *L. camara.*
Figure S3: MS spectra for cis-β-copaene detected in *L. camara.*
Figure S4: MS spectra for Cadina-1(10),4-diene detected in *L. camara.*
Figure S5: MS spectra for α-Pinene detected in *E. globulus.*
Figure S6: MS spectra for Eucalyptol detected in *E. globulus.*
Figure S7: MS spectra for Isopinocarveole detected in *E. globulus.*
Figure S8: MS spectra for Pinocarvone detected in *E. globulus.*

Table S1. Retention times, MS ion fragments and identified compounds for *L. camara* and *E. globulus* essential oils.

RT	Identified Compound[a]	Peak Area %	Base peak, Molecular ion	Other Key Fragments
		L. camara		
5.30	α-thujene	4.02	93,136	77,105,121
6.08	α-Pinene	13.83	93,136	77,105,121
6.29	Camphene	1.99	93,136	69,79,107,121
7.53	Sabinene	1.55	93,136	68,79,107,121
7.81	β-Pinene	2.73	93,136	67,79,105,121
8.48	β-Myrcene	1.47	93,136	65,77,105,121
15.20	Terpinene-4-ol	1.25	93,154	71,111,121
30.57	β-elemene	4.08	93,204	67,81,107,121,147,189
33.56	Caryophyllene	30.99	133,204	79, 91,105,120,147,189
33.93	Bicyclogermacrene	4.11	121,204	67, 79, 93, 107, 133,161,189
34.13	Alloaromadendrene	1.54	161,204	67,79,91,105,119,133
35.59	(E)-β-Farnesene	2.26	69,204	55,79,93,105,120,133,161
36.24	α-humulene	2.18	93,204	67,80,107,121,147,161
37.78	γ - Curcumene	1.19	119,204	55,69,77,93,105,133,161
38.38	cis-β-copaene	5.02	161,204	67,81,91,105,119,133
39.56	γ - muurolene	3.04	161,204	55,67,81,91,105,119,133,189
41.32	Cadina-1(10),4-diene	5.92	161,204	55,81,105,119,134,189
46.82	Caryophyllene oxide	1.33	79, 220	55,69,79,91,107,121, 131,161,187
49.08	unknown*	1.98	85,220	55,67,79,91,105,121, 131,159,187
51.56	δ-Cadinene	1.36	161,204	55,81,91,105,119,134,189
52.59	β-vatirenene	1.7	159,202	55,79,91,105,131,145,187
		E. globulus		
13.21	α-Pinene	9.09	93,136	53,67,77,105,121
21.23	p-cymene	1.17	119,134	65,77,91,103
21.50	m-cymene	1.86	119,134	77,91,103
22.38	Eucalyptol	52.77	81,154	55,71,93,108,125,139
33.30	Isopinocarveole	16.72	92,152	55,70,83,109,119
35.53	Pinocarvone	10.93	81,150	53,69,91,108,122,135
36.30	Endo-Borneol	1.65	95,154	55,67,79,110,121, 136
38.56	α-Terpineol	1.14	93,154	59,67,79,93,107,121,136

a Only constituents with relative abundance ≥1% are included.

* Mass spectral data did not allow reliable identification.

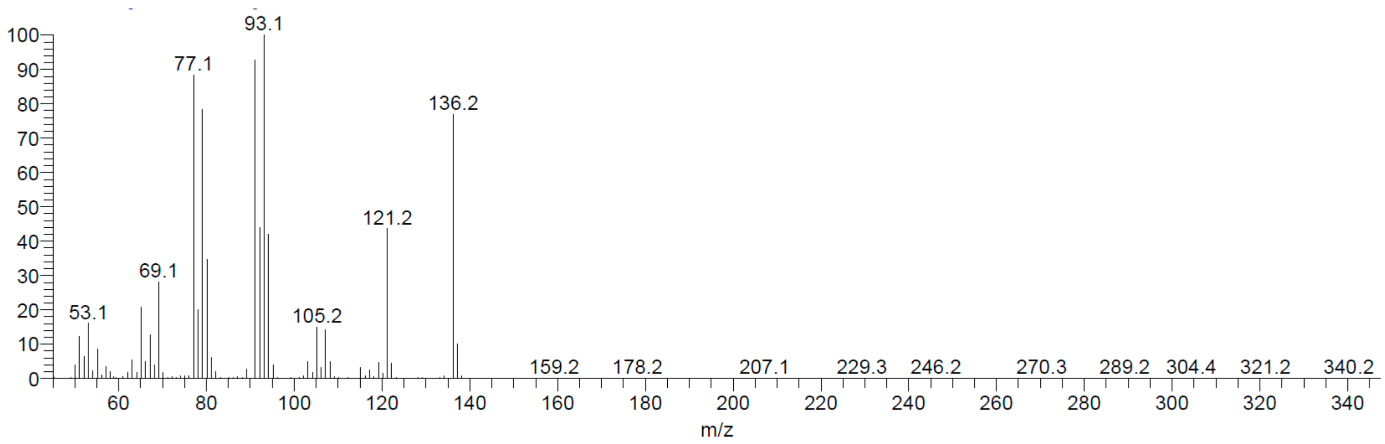

Figure S1: MS spectra for α-Pinene detected in *L. camara.*

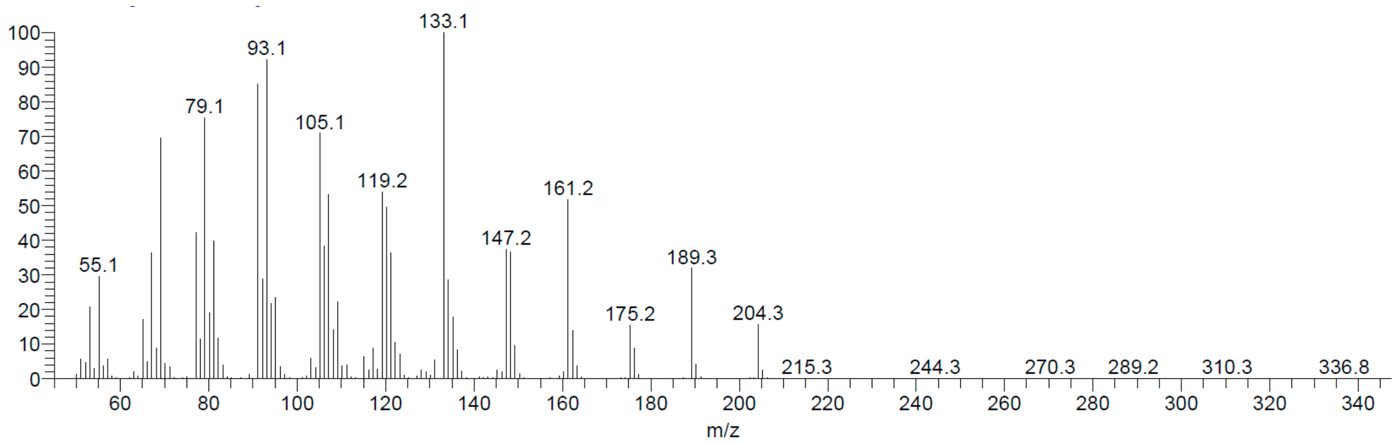

Figure S2: MS spectra for Caryophyllene detected in *L. camara.*

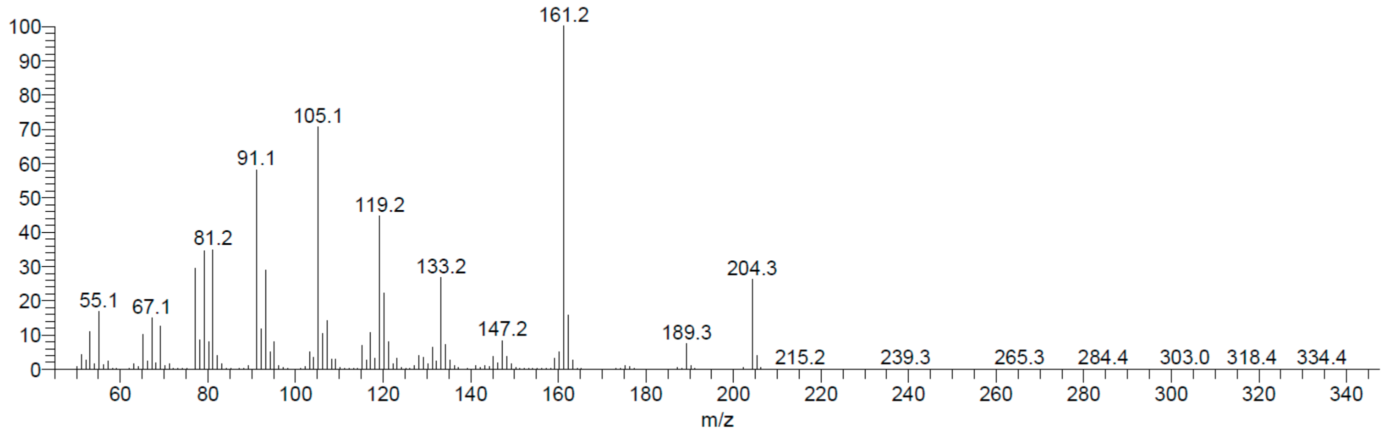

Figure S3: MS spectra for cis-β-copaene detected in *L. camara.*

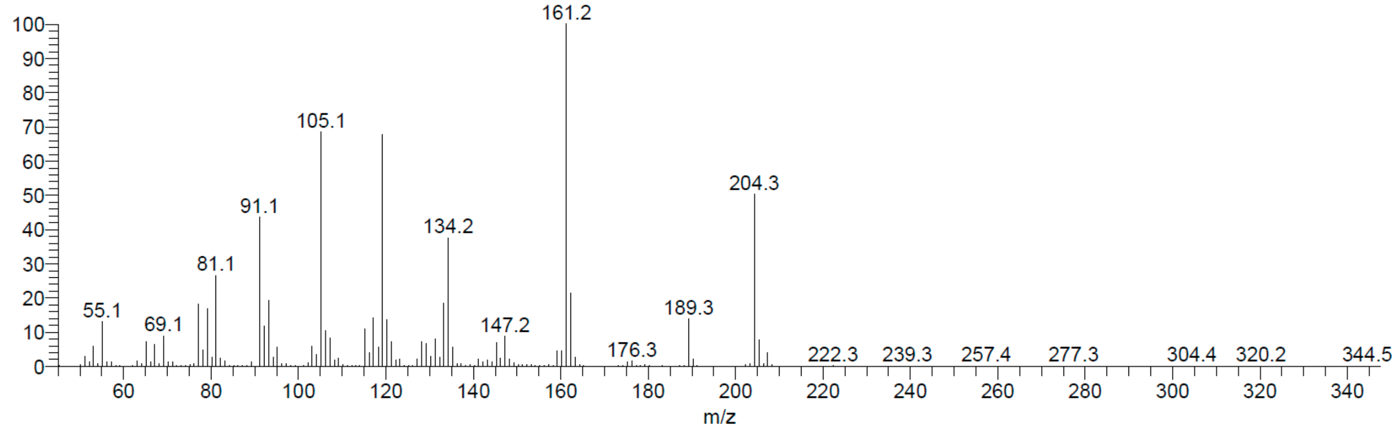

Figure S4: MS spectra for Cadina-1(10),4-diene detected in *L. camara.*

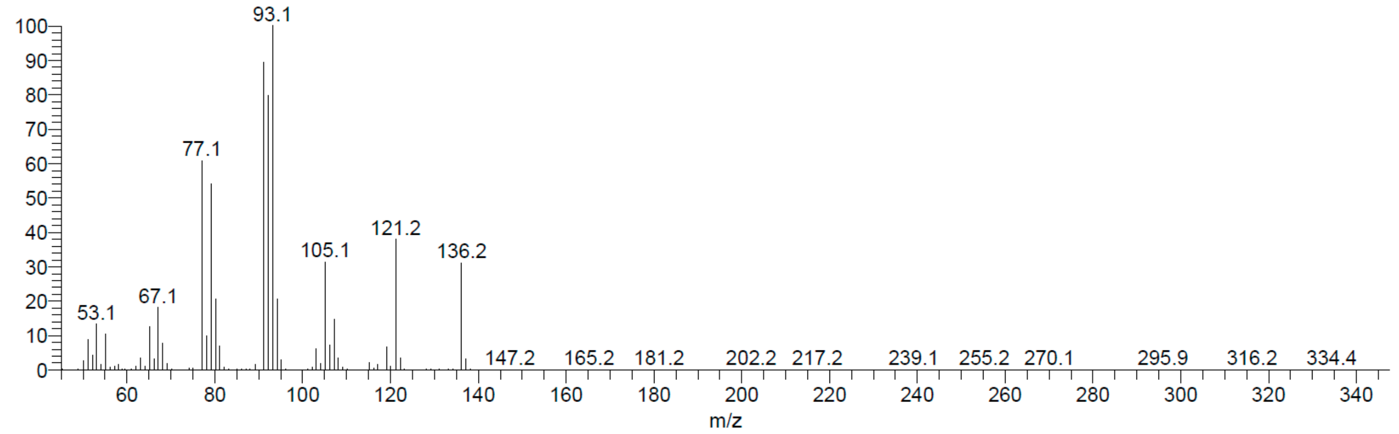

Figure S5: MS spectra for α-Pinene detected in *E. globulus.*

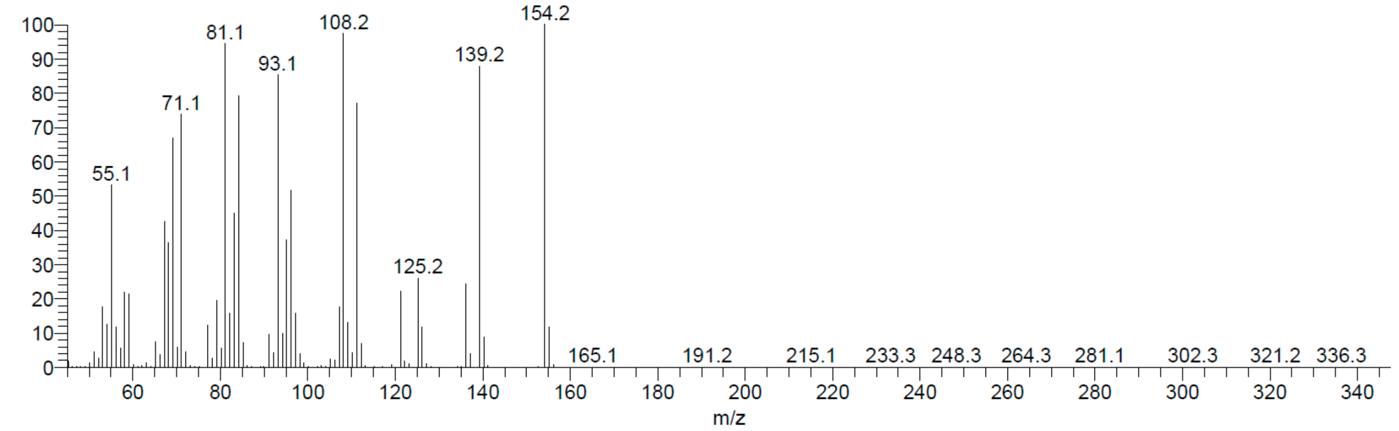

Figure S6: MS spectra for Eucalyptol detected in *E. globulus.*

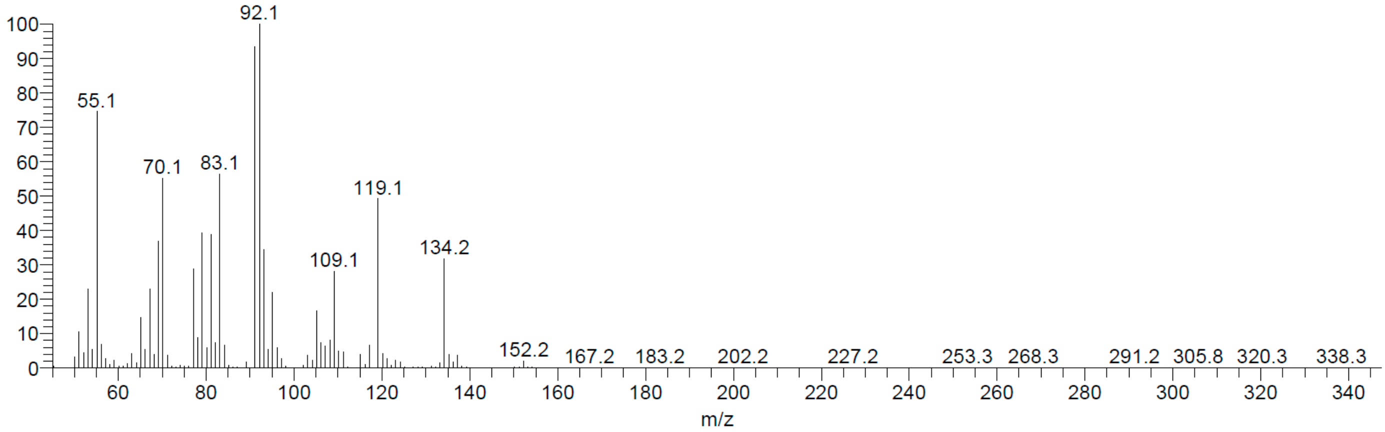

Figure S7: MS spectra for Isopinocarveole detected in *E. globulus*.

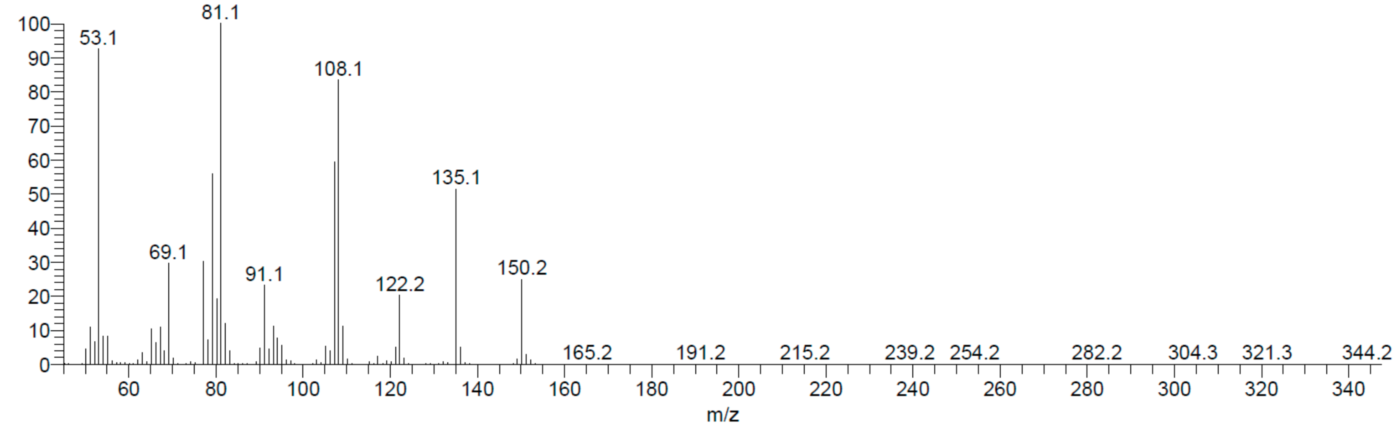

Figure S8: MS spectra for Pinocarvone detected in *E. globulus*.
